# Self-Consistent Enhanced S/D Tunneling Implementation in a 2D MS-EMC Nanodevice Simulator

**DOI:** 10.3390/mi12060601

**Published:** 2021-05-22

**Authors:** Cristina Medina-Bailon, José Luis Padilla, Carlos Sampedro, Luca Donetti, Vihar P. Gergiev, Francisco Gamiz, Asen Asenov

**Affiliations:** 1 Nanoelectronics Research Group, Departamento de Electrónica y Tecnología de Computadores, Universidad de Granada, 18071 Granada, Spain; jluispt@ugr.es (J.L.P.); csampe@ugr.es (C.S.); donetti@ugr.es (L.D.); fgamiz@ugr.es (F.G.); 2 Device Modelling Group, School of Engineering, University of Glasgow, Glasgow G12 8LT, UK; Vihar.Georgiev@glasgow.ac.uk (V.P.G.); Asen.Asenov@glasgow.ac.uk (A.A.)

**Keywords:** direct source-to-drain tunneling, tunneling probability, Landauer formalism, multi-subband ensemble Monte Carlo, non-equilibrium Green’s functions, DGSOI, FinFET

## Abstract

The implementation of a source to drain tunneling in ultrascaled devices using MS-EMC has traditionally led to overestimated current levels in the subthreshold regime. In order to correct this issue and enhance the capabilities of this type of simulator, we discuss in this paper two alternative and self-consistent solutions focusing on different parts of the simulation flow. The first solution reformulates the tunneling probability computation by modulating the WKB approximation in a suitable way. The second corresponds to a change in the current calculation technique based on the utilization of the Landauer formalism. The results from both solutions are compared and contrasted to NEGF results from NESS. We conclude that the current computation modification constitutes the most suitable and advisable strategy to improve the MS-EMC tool.

## 1. Introduction

In the field of transport simulation in ultrascaled nanostructures, and in order to preserve the prediction capabilities of simulation tools, it is mandatory to account for phenomena that were not relevant in previous technological nodes. In particular, incorporating quantum mechanical tunneling into semiclassical models has become popular due to its modular implementation and its reduced computational time compared to full quantum simulation approaches. One example of this efficient technique is the integration of a source-to-drain tunneling (S/D tunneling) module into a 2D Multi-Subband Ensemble Monte Carlo (MS-EMC) simulator [1,2].

This type of tunneling allows carriers to cross the channel barrier passing from the source directly to the drain. As a result, the subband potential profile might change, which would lead to an increase in the subthreshold current, eroding the electrostatic control of the gate. S/D tunneling is traditionally considered as a scaling limit in ballistic Non-Equilibrium Green’s Function (NEGF) calculations [3], which distorts the MOSFET operation at channel lengths around 3 nm [4].

In this work, we present an enhanced implementation of the S/D tunneling estimation in the MS-EMC simulator based on a two-fold approach. First, a more robust current computation, and second, a modification of the traditional WKB tunneling probability. The simulation results that we present in this paper are doubly tested by comparing them with independent series of data obtained from two different sources. One source is the previous S/D tunneling implementation inside the MS-EMC tool [1,2], and the other is the modular and open-source TCAD semiconductor device simulator called Nano-Electronic Simulation Software (NESS) [5,6,7,8].

Regarding the results obtained from NESS and utilized in this work, they were generated by means of the 2D approximation [2] of the coupled mode-space NEGF solver included in it. This NEGF module allows for the quantum treatment of charge transport so that quantum mechanical phenomena such as tunneling, coherence, and particle-particle (wave-wave) interactions can be accounted for in mesoscopic and nanoscale device structures. It is worth noting that, although the results from NESS that we consider herein only assume ballistic transport, this simulator would also allow us to compute acoustic and/or optical phonon scattering in order to describe electron–phonon (e–ph) interactions within the self-consistent Born approximation (SCBA). This choice of ballistic comparison between both simulators has been made in order to avoid potential discrepancies between them derived from their different scattering implementations. In other words, a pure ballistic analysis allows a highly trustworthy comparison between S/D tunneling models in MS-EMC and NESS.

This paper is organized as follows. In Section 2, we describe the analyzed devices along with the MS-EMC capabilities to account for S/D tunneling. We also present the proposed strategies for the enhancement of the simulator. Section 3 is devoted to the results, assessing the suitability of each of the proposed improvements for the MS-EMC tool. Finally, the main conclusions of our study are drawn in Section 4.

## 2. Simulation Framework and Device Structures

### 2.1. Description of the Simulated Devices

**Figure 1:**
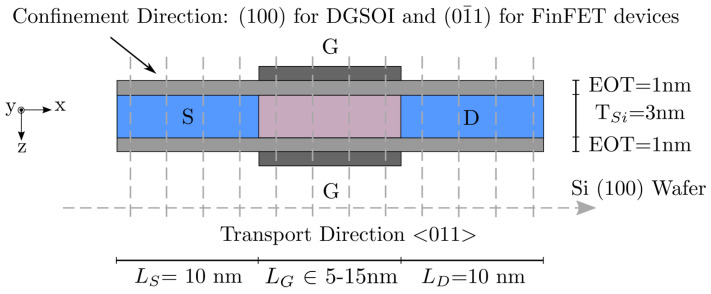
Schematic illustration of the Si DGSOI and FinFET structures with $L_{G}$ ranging from 5 nm to 15 nm. The rest of the parameters remains fixed: source length ($L_{S}$ = 10 nm), drain length ($L_{D}$ = 10 nm), Si thickness ($T_{Si}=3$ nm), and Equivalent Oxide Thickness (EOT =1 nm). The confinement direction for these devices on standard wafers [100] changed from (100) for DGSOI to ($0\bar{1}1$) for FinFET, whereas the transport direction <011> was the same for both. 1D Schrödinger equation was solved for each grid point in the transport direction, and BTE was solved by the MC method in the transport plane.

In Figure 1, we present the structure and description corresponding to the the planar Double-Gate Silicon-On-Insulator (DGSOI) structure and vertical Si FinFET to which we applied our enhanced S/D tunneling description. For comparison with the NEGF-NESS tool, ballistic transport and bulk effective masses were considered, as indicated above. Additionally, the gate work functions were appropriately modified for each device so as to provide the same threshold current ($I_{\mathrm{TH}}$). $m_{\mathrm{bulk}}$ values were estimated by DFT in Quantum ATK tool of Synopsys [9] in accordance with [2] to obtain more realistic conduction band profiles in nanoscaled devices.

In terms of subbands, the lowest energy was attained for $\Delta_{2}$ in the planar transistor, whereas it shifted to $\Delta_{4}$ in the vertical one. It is important to notice that, although the FinFET is a 3D structure and our simulation approach is 2D, it has been shown that FinFETs with fin heights much larger than their corresponding thicknesses show similar behavior in all transport regimes when using 2D and 3D simulations [10].

Gate lengths for the devices under study were designed to range between 5 nm and 15 nm. The rest of the technological parameters remained constant: channel thickness ($T_{\mathrm{Si}}=3$ nm), SiO_2_ gate oxide (EOT =1 nm) and metal gate work function (4.385 eV).

### 2.2. General Overview of the 2D MS-EMC Tool

The simulation approach of the 2D MS-EMC tool employed in this work [11] combines a semiclassical implementation along with a decoupled mode-space quantum transport conception [12]. The simulator solved the Schrödinger equation in the confinement direction for the the discretized slices, and the Boltzmann Transport Equation (BTE) in the transport plane (see Figure 1). Both equations were coupled through the 2D Poisson equation in the whole 2D simulation domain to maintain the self-consistency of the solution.

This tool has been widely used in different scenarios and, in particular, for the study and assessment of different types of tunneling [13] in ultrascaled devices. The implementation and inclusion of these tunneling mechanisms into the 2D MS-EMC simulator was successfully carried out thanks to the modular design of the tool. This means that each tunneling phenomenon was incorporated through a separate module that treats it as a differentiated transport mechanism. This modular strategy does not represent an increase in the computational time in comparison to purely quantum simulators and, in return, allows each of the different mechanisms to be switched on and off depending on our particular volition and the considered scenario. It becomes clear that such development offers a wide range of possibilities in terms of comparison between various tunneling types, and opens the door for thorough analyses focusing on any of them.

### 2.3. S/D Tunneling Implementation inside the 2D MC-EMC Tool

**Figure 2:**
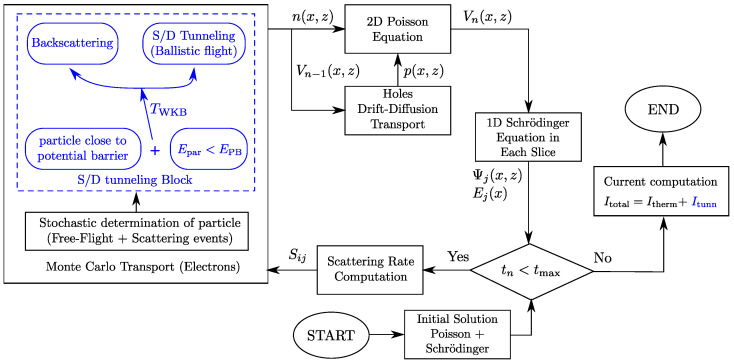
Flowchart of the S/D tunneling computation inside the MS-EMC simulator through its corresponding add-on module. In the diagram, *x* and *z* are the transport and confinement directions, respectively. n(x,z) and p(x,z) stand for the electron and hole concentrations, respectively. V(x,z) is the potential profile, and $S_{ij}$ is the scattering rates. $E_{j}\left(x\right)$ is the energy profile of the *j*th subband, and $\Psi_{j}\left(x,z\right)$ represents the corresponding eigenfunctions. The subscript *n* stands for the iteration number, and $t_{n}$ together with $t_{\max}$ is the successive time steps and the final time, respectively.

Figure 2 shows a detailed flow diagram of the S/D tunneling implementation into the 2D MS-EMC simulation [1]. In the diagram, $E_{\mathrm{par}}$ represents the particle energy, $E_{\mathrm{PB}}$ is the energy of the potential barrier to be crossed, and $T_{\mathrm{WKB}}$ is the transmission probability through that barrier for a given energy. As suggested by the subscript, this tunneling probability utilizes the WKB approximation, which reads as

(1) $$T_{\mathrm{WKB}}\left(E_{x}\right)=\exp\left[-\frac{2}{\hbar }\int_{a}^{b}\;\sqrt{2m_{x}\left(E_{i}\left(x\right)-E_{x}\right)}\;dx\right],$$

where *a* and *b* are the limits of the tunneling path, $m_{x}$ is the tunneling effective mass of the electron, and $E_{x}$ is the total energy in the transport plane considering only the projection of the kinetic energy in the direction that faces the potential barrier. In that sense, it is important to clarify that the *x* subscript of $m_{x}$ and $E_{x}$ does not refer to any particular spatial position but rather represents an explicit reminder of the transport direction itself.

With the implementation of S/D tunneling depicted in Figure 2 and the computation of the current using the conventional method of counting the number of particles moving inside a previously fixed space window, the simulation results obtained showed that the current contribution of this quantum phenomenon proved to be higher compared to the levels reported by a NEGF approach [14]. This discrepancy turned out to be particularly noticeable as gate lengths decreased in the analyzed devices. One plausible strategy to accommodate the MS-EMC results to those provided by NEGF could be to focus on the transmission probability and contemplate the hypothesis that the utilization of the WKB approximation overestimates the number of superparticles experiencing S/D tunneling. Another strategy might be to reformulate the way in which the tunneling current is computed in the MS-EMC code dissociating it from the aforementioned conventional method. Both standpoints are hereinafter discussed. The first one leads to the proposal of a modified tunneling probability, whereas the second results in the utilization of the Landauer formula for the tunneling current computation.

#### 2.3.1. Standpoint 1. Reformulation of the Tunneling Probability

An alternative for defining an accurate S/D tunneling probability to be employed in the 2D MS-EMC simulator can be found in [2], using a non-local formulation based on the extension of a previous work [15]. As proposed, the probability could be redesigned in the following way:

(2) $$T_{\mathrm{DT}}\left(E_{x}\right)=\frac{\Delta_{y}}{2\sqrt{\pi }}{\left[\hbar \int_{a}^{b}\frac{dx}{\sqrt{2m_{x}\left(E_{i}\left(x\right)-E_{x}\right)}}\right]}^{-1/2}\cdot T_{\mathrm{WKB}}\left(E_{x}\right),$$

where $\Delta_{y}$ is the mesh spacing in the direction normal to transport. However, since this direction is not taken into account in the 2D simulation, $\Delta_{y}$ needs to be externally estimated and fixed in advance so that it will not depend either on the type of device or the peculiarities of the simulation.

At first sight, this expression and the presence of $\Delta_{y}$ pose two major concerns. The first is procedural, and refers to the necessary external precalibration of $\Delta_{y}$ which harms the self-consistency of the MC operation. The second is conceptual and makes reference to the form of Equation (2), which does not guarantee a probability lying within the range [0–1]. Moreover, from a purely theoretical point of view, sharp barrier profiles (such as an inverted triangular well) would make the term between square brackets amount to zero, thus depriving $T_{\mathrm{DT}}$ of any meaning. However, this drawback is not realistic in practical terms since the energy barriers typically feature smooth profiles leading to finite values of $T_{\mathrm{DT}}$. In any case, the theoretical risk still lies beneath.

**Figure 3:**
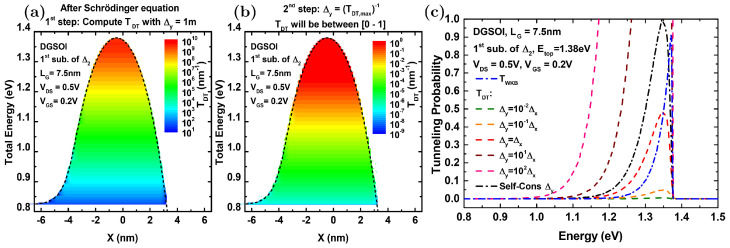
Example of the procedure that restores self-consistency in the MS-EMC simulation by means of $T_{\mathrm{DT}}$ by defining a suitable value for Δy that also works as a normalization factor for the probability. (**a**) After solving the Schrödinger equation in each time step, we computed $T_{\mathrm{DT}}\left(E_{x}\right)$ taking Δy=1 m. (**b**) We took the maximum of this initial estimation of $T_{\mathrm{DT}}$ and defined Δy as its inverse, which led to $T_{\mathrm{DT}}$ probabilities between 0 and 1. (**c**) We plotted $T_{\mathrm{DT}}$ as a function of the total energy considering different choices of Δy (self-consistent and dependent on Δx).

If one neglects this theoretical issue and limits to the practical domain where the energy barriers of interest provide finite values of $T_{\mathrm{DT}}$, one might recover self-consistency (recall the first concern listed above) by using Δy as a normalization parameter that restores the maximum value of the tunneling probability to one. The procedure consists of two successive steps illustrated with an example in Figure 3. At first, after solving the Schrödinger equation in each time step, we computed $T_{\mathrm{DT}}\left(E_{x}\right)$ taking Δy=1 m. This led to a function that typically increased with $E_{x}$ for each subband (Figure 3a). Immediately thereafter, we took the maximum of this initial estimation of $T_{\mathrm{DT}}$ and defined Δy as its inverse (Figure 3b). These calculated $T_{\mathrm{DT}}$ probabilities were then between 0 and 1 (as illustrated by the color scale in Figure 3b). We also considered and plotted, for comparison, the $T_{\mathrm{DT}}$ curves corresponding to alternative and pre-established values of Δy (Figure 3c). It is obvious that, for those predefined values of Δy, the maximum values of their counterpart $T_{\mathrm{DT}}$ curves do not correspond to 1.

#### 2.3.2. Standpoint 2. Tunneling Current Computation by Means of the Landauer Formula

The second approach to accommodate the results from the 2D MS-EMC simulator to those reported by NEGF focuses on reformulating the way in which the tunneling current is computed. This point of view assumes that the utilization of the WKB approximation for the tunneling probability estimation holds as valid and does not need to be modified.

The traditional procedure to estimate the current in the MC code employs the technique of counting the number of particles inside a previously fixed spatial window across the device, whose size is tightly conditioned by the length of the channel, and multiplying them by their velocity. This corresponds to $v_{x}\cdot \mathrm{epp}$, where epp stands for the number of electrons per particle. Considering this, and given the restricted number of particles experiencing S/D tunneling, the choice of the most suitable window for computing their current contribution needs careful assessment (especially for subthreshold current levels). Otherwise, if the window is not precisely adjusted in advance, the procedure might lead to inaccurate results [14]. The necessity of previous knowledge regarding the expected shape and thickness of the barrier for an appropriate choice of spatial window indicates that this traditional method for current computation is not the most advisable in the presence of this kind of tunneling.

An appealing alternative for computing the current in the presence of such S/D tunneling processes lies in the so called Landauer approach [16,17], which conceives the current across a certain spatial region as if it could be expressed in terms of the probability of an electron transmitting through it. From this perspective, even thermionic current over the barrier could be modeled this way by simply fixing the transmission probability to one for energies above the maximum of the barrier.

Assuming this point of view, the computation of the total S/D current could be entirely unified so that in the following expression

(3) $$I_{\mathrm{total}}=I_{\mathrm{therm}}+I_{\mathrm{tunn}},$$

the term corresponding to $I_{\mathrm{therm}}$ is computed similarly to $I_{\mathrm{tunn}}$ but with “tunneling” probability equal to one for energies above the barrier. Therefore, under the Landauer approach, the most general expression for the current (that of $I_{\mathrm{tunn}}$) reads as

(4) $$I_{\mathrm{tunn}}=\frac{q}{h}\frac{2}{\pi }\int_{-\infty }^{\infty }|F_{2D}\left(E_{x}-E_{FS}\right)-F_{2D}\left(E_{x}-E_{FD}\right)|\;T_{\mathrm{WKB}}\left(E_{x}\right)\;dE_{x},$$

where *q* is the electron charge, *h* is the Planck constant, and $E_{FS(D)}$ is the Fermi level at the source (drain) contact. It is worth noting how the transmission probability is modeled by means of the WKB approximation, as indicated above in Equation (1). $F_{2D}$ is the 2D Fermi-Dirac function given by

(5) $$F_{2D}\left(E^{\prime }\right)=\int_{-\infty }^{\infty }f\left(E^{\prime }+\frac{\hbar^{2}k_{y}^{2}}{2m_{y}}\right)\;dk_{y},$$

with *f* the Fermi-Dirac distribution expressed as

(6) $$f\left(E\right)=\frac{1}{1+e^{E/kT}}$$

## 3. Results

### 3.1. Comparison between the Different Implementations of the S/D Tunneling Probability in MS-EMC and the Simulation Results from NEGF

In this section, we describe the utilization of the standard current computation method based on counting the particles and multiplying them by their corresponding velocity, and the analysis of the impact of adopting the tunneling probability $T_{\mathrm{DT}}$ for describing the S/D tunneling processes. Results are compared for various Δy values (namely, the one that restores self-consistency and others arbitrarily chosen with respect to the mesh spacing in the *x* direction), as well as for the case where the transmission probability is estimated by means of the WKB approximation.

**Figure 4:**
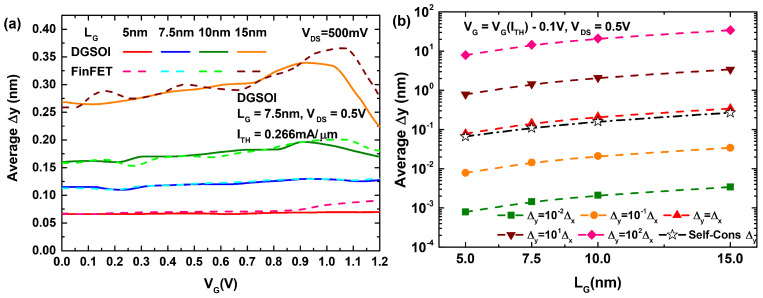
(**a**) Behavior of the average self-consistent Δy estimation as a function of $V_{G}$ for the DGSOI and FinFET with $L_{G}$ ranging from 5 nm to 15 nm. (**b**) Effect of gate length increase on the different Δy definitions (self-consistent and dependent on Δx).

$V_{\mathrm{DS}}$ was fixed to 0.5 V in all cases unless otherwise specified. If we observe the behavior of the self-consistent Δy value when the gate voltage varies, Figure 4a shows that for reduced gate lengths below 15 nm (those typically most affected by S/D tunneling) this parameter proves to be almost constant, featuring only very slight variations for both considered devices. For ease of understanding, since the self-consistent Δy was computed for each MC iteration, the results displayed in our curves correspond to average values over a number of iterations. Figure 4b illustrates the impact on the different Δy values when we increased the gate length and for a fixed gate voltage below the threshold current level. The rising pattern for the predefined values of Δy dependent on Δx (the horizontal meshing distance) is explained if one notes that our simulations employed a fixed number of mesh points in the transport direction, which caused Δx to increase as $L_{G}$ increased. It is interesting to observe how the self-consistent Δy parameter almost fits with the value of Δx in the last figure.

**Figure 5:**
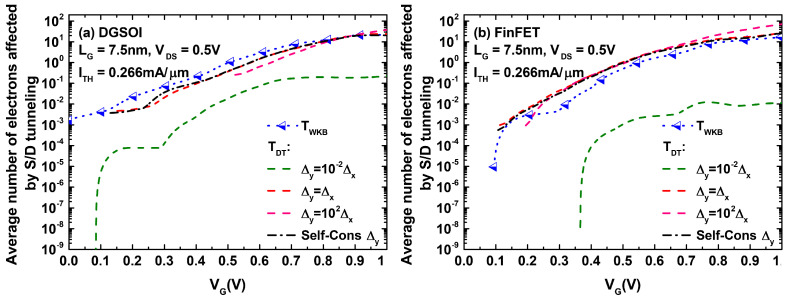
Average number of electrons (in arbitrary units) affected by S/D tunneling as a function of $V_{G}$ in the DGSOI (**a**) and the FinFET (**b**) with $L_{G}=7.5$ nm. The results compare the utilization of the WKB probability with respect to $T_{\mathrm{DT}}$ with different choices of Δy (self-consistent and predefinedly dependent on Δx).

As immediately understood from Equation (2), different choices of Δy have a direct effect on the resulting $T_{\mathrm{DT}}$ value employed in our simulations. This, in turn, would affect the number of superparticles affected by S/D tunneling. Figure 5 shows the average number of electrons tunneling from the source to drain as a function of the gate biasing when both devices have a gate length of $L_{G}=7.5$ nm and feature the same threshold current. In some cases, the number of tunneling electrons computed using $T_{\mathrm{DT}}$ exceeded their WKB counterpart. This is particularly noticeable for the FinFET structure.

**Figure 6:**
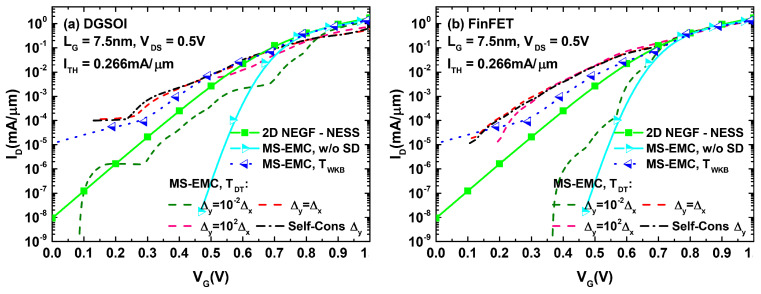
$I_{D}-V_{G}$ characteristics for the DGSOI (**a**) and FinFET (**b**) with $L_{G}=7.5$ nm employing different methods for computing S/D tunneling. On one hand, we display the set of curves obtained from the MS-EMC tool using $T_{\mathrm{WKB}}$ and $T_{\mathrm{DT}}$ (with various Δy choices) and, on the other, the curve calculated using the NEGF formalism of NESS.

**Figure 7:**
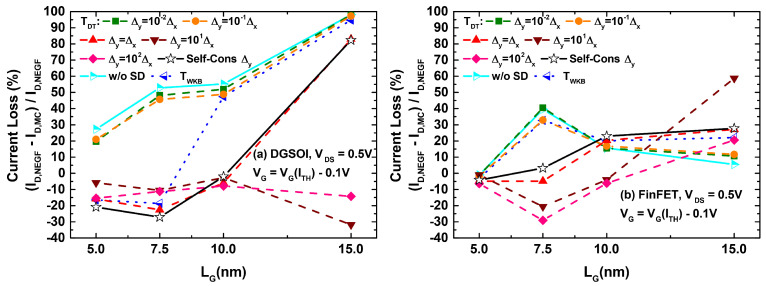
Subthreshold current loss as a function of $L_{G}$ in the DGSOI (**a**) and FinFET (**b**) between NESS and MS-EMC with the different implementations of the tunneling probability in the Latter.

These results could then be extrapolated for analyzing their effect on the $I_{D}-V_{\mathrm{GS}}$ characteristics of both devices. The corresponding plots are displayed in Figure 6. A close inspection of the different curves for both devices indicates that the main differences between the set of curves corresponding to S/D tunneling computation in MC and the one obtained from NEGF calculations arose for current levels below the threshold reference. This behavior discrepancy disappeared when the devices entered the ON regime. With the objective of quantifying the current deviation between MS-EMC and NESS in the subthreshold regime, we selected for each analyzed curve a gate voltage 0.1 V below that corresponding to $I_{\mathrm{TH}}$. For this gate bias, we depict in Figure 7 the current loss defined as $\left(I_{\mathrm{NEGF}}-I_{\mathrm{MS}-\mathrm{EMC}}\right)/I_{\mathrm{NEGF}}$. We observed how, for values of $\Delta y\leq 10^{-1}\Delta x$, the reported losses approached those corresponding to the situation without tunneling. On the other hand, for gate lengths for which S/D tunneling became significant ($L_{G}\leq 10$ nm), the cases with Δy>Δx exhibited a negative current loss or, in other words, an overestimation of the tunneling contribution in MC ($I_{\mathrm{MS}-\mathrm{EMC}}>I_{\mathrm{NEGF}}$). The self-consistent choice of Δy (leading to Δy≈Δx) reports the closest resemblance to the current levels obtained from NEGF. Nevertheless, the percentage variations remained noticeable in this case.

These results suggest that the most appropriate approach for optimizing the S/D tunneling implementation in the MS-EMC simulator might be the other strategy based on the modification of the tunneling current computation through the Landauer approach. This is indeed confirmed in the next section.

### 3.2. Comparison between the Different Current Computation Strategies in MS-EMC and the Simulation Results from NEGF

As detailed in Section 2.3.2, the second approach to modify the implementation of S/D tunneling in the MS-EMC tool involves the variation of the methodology for estimating the tunneling current. By utilizing the Landauer formalism, we may additionally benefit from the simplification of considering the thermionic current through the barrier as a particular type of crossing event with a transmission probability equal to one. This interpretation justifies the utilization of Equation (4) for both transport mechanisms, leading to a procedural unification that constitutes an elegant and sound proposal for the current computation in MS-EMC in the presence of tunneling events between source and drain.

Given that this section aims to quantify the impact of varying the current computation technique, we isolate its effect from the modification of the tunneling probability estimation analyzed in Section 3.1. Therefore, whenever the Landauer formalism is employed in any of the curves shown below utilizing the MC simulator, those results correspond to the utilization of the standard WKB tunneling probability.

**Figure 8:**
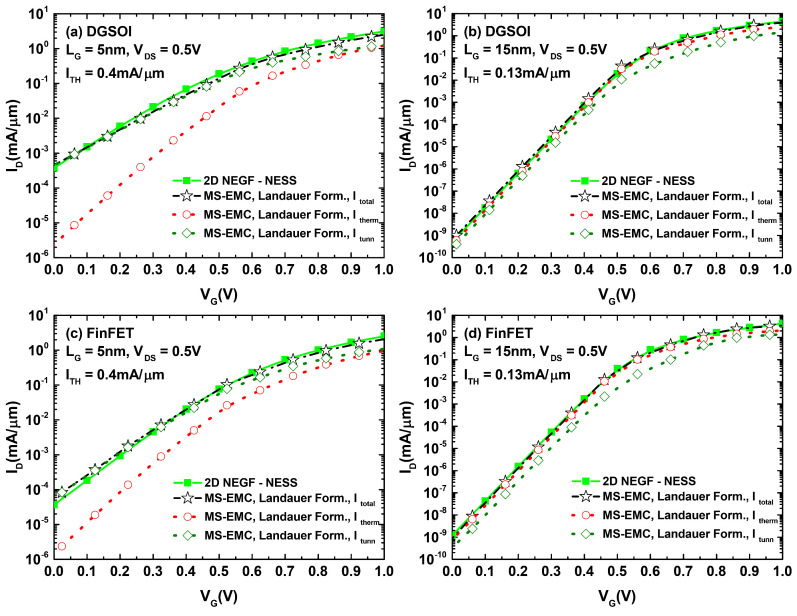
$I_{D}-V_{G}$ characteristics for the DGSOI and FinFET with the shortest and longest gate lengths considered in our work, respectively: (**a**) DGSOI with $L_{G}=5$ nm, (**b**) DGSOI with $L_{G}=15$ nm, (**c**) FinFET with $L_{G}=5$ nm, and (**d**) FinFET with $L_{G}=15$ nm. The plots compare the results from NEGF with those from MS-EMC employing the Landauer approach for the current computation. The total current reported from MS-EMC was also broken down into its tunneling and thermionic contributions.

The first comparison performed is shown in Figure 8, where we show the excellent resemblance between the total current computed with MS-EMC and that obtained from NEGF. This agreement is verified for the two analyzed structures (DGSOI and FinFET) and within the considered range of gate lengths. As to illustrate this, we simply depict the two extreme values of $L_{G}$, namel, y 5 nm and 15 nm.In the set of plots of Figure 8, we also explicitly broke down the two components ($I_{\mathrm{therm}}$ and $I_{\mathrm{tunn}}$) of MS-EMC that add up to $I_{\mathrm{total}}$ showing the relative importance of each one depending on the gate biasing conditions. It is particularly noticeable how, starting from the situation where $I_{\mathrm{therm}}$ prevailed over $I_{\mathrm{tunn}}$ for 15 nm, the relative importance of $I_{\mathrm{tunn}}$ with respect to $I_{\mathrm{therm}}$ increased gradually as the channel length shrank.

**Figure 9:**
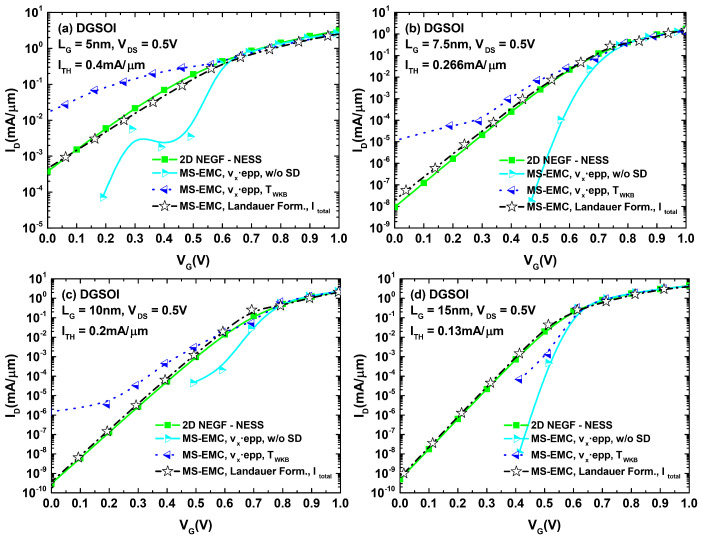
$I_{D}-V_{G}$ characteristics for the DGSOI device with (**a**) $L_{G}=5$ nm, (**b**) $L_{G}=7.5$ nm, (**c**) $L_{G}=10$ nm, and (**d**) $L_{G}=15$ nm. The displayed current curves correspond to: NEGF, MS-EMC with the standard current computation (with and without S/D tunneling), and MS-EMC using the Landauer approach.

**Figure 10:**
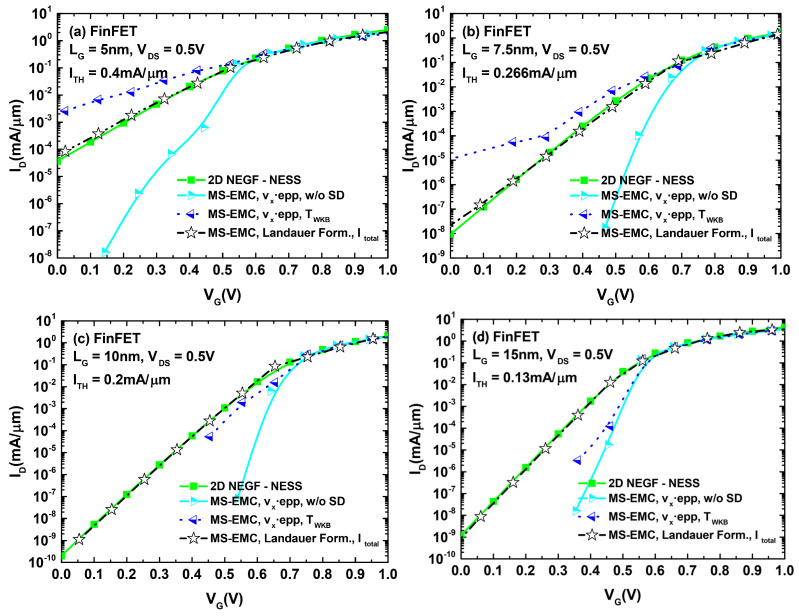
$I_{D}-V_{G}$ characteristics for the FinFET with different gate lengths: (**a**) $L_{G}=5$ nm, (**b**) $L_{G}=7.5$ nm, (**c**) $L_{G}=10$ nm, and (**d**) $L_{G}=15$ nm. Analogously to the DGSOI, the displayed current curves correspond to: NEGF, MS-EMC with the standard current computation (with and without S/D tunneling), and MS-EMC using the Landauer approach.

In light of Figure 9 and Figure 10, we can confirm how the utilization of the Landauer current calculation provided a significant improvement of the simulated subthreshold current levels compared to those arising from the standard MC current estimation. A number of simulations were performed for the DGSOI device (Figure 9) for different gate lengths. They showed that, as the importance of S/D tunneling increased ($L_{G}\leq 10$ nm), the standard MC approach systematically led to a current overestimation in the subthreshold regime. Moreover, the employment of the Landauer formula not only provided current levels fitting those coming from NEGF, but also retrieved the characteristic expected subthreshold linearity of the curves in logarithmic scale. These conclusions from the DGSOI structure can be perfectly extrapolated to the FinFET by inspecting Figure 10. For this device, it can be seen how the current overestimation from the standard computation technique started to show up at $L_{G}=7.5$ nm and below. All in all, the results contained in Figure 8, Figure 9 and Figure 10 constitute an explicit demonstration of how robust and advisable the Landauer approach proves to be when incorporated to the MS-EMC simulator to compute the current in the subthreshold regime.

**Figure 11:**
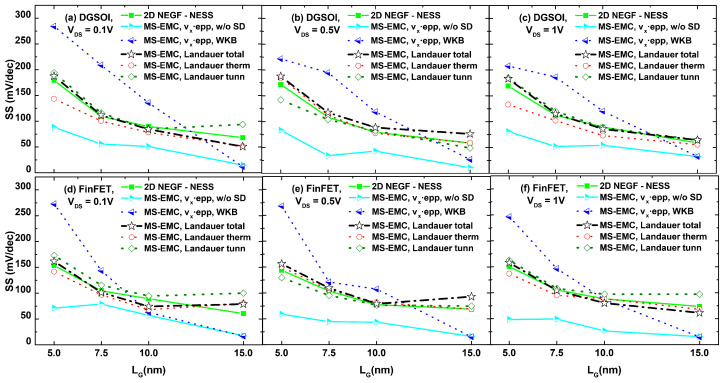
Subthreshold swing values (SS) as a function of the gate length for the DGSOI and FinFET considering different drain voltages: (**a**) DGSOI with $V_{\mathrm{DS}}=0.1$ V, (**b**) DGSOI with $V_{\mathrm{DS}}=0.5$ V, (**c**) DGSOI with $V_{\mathrm{DS}}=1$ V, (**d**) FinFET with $V_{\mathrm{DS}}=0.1$ V, (**e**) FinFET with $V_{\mathrm{DS}}=0.5$ V, and (**f**) FinFET with $V_{\mathrm{DS}}=1$ V. Current computation corresponds to the different techniques analyzed in our work: NEGF, MS-EMC with the standard approach (with and without S/D tunneling), and MS-EMC incorporating Landauer.

A detailed assessment of the subthreshold behavior of the current curves displayed in Figure 8, Figure 9 and Figure 10 can be found in Figure 11 through the extraction of their corresponding subthreshold swing (SS) values. These swing values were computed focusing on the part of the curves whose current levels lie between $10^{-3}$ mA/μm and $10^{-2}$ mA/μm. It is clear that, in each plot of Figure 11, the lowest limit for the SS was determined in absence of tunneling. The SS curves exhibited an increasing trend as the gate length was reduced, thus quantifying the impact of S/D tunneling when this phenomenon became more relevant. The current overestimation from the standard current calculation in MS-EMC is apparent from these plots, leading to a more pronounced swing degradation as the gate length decreases. SS values reported by MS-EMC utilizing the Landauer approach are in very close agreement with those corresponding to NEGF, especially for ultrascaled devices.

## 4. Conclusions

In this work, we discussed two alternative solutions oriented to enhance the implementation of S/D tunneling in an MS-EMC simulator aiming to eradicate the subthreshold current overestimation reported when this type of tunneling is analyzed in MC. The first solution focuses on reformulating the tunneling probability computation by modulating the WKB approximation, which gives rise to the so called $T_{\mathrm{DT}}$ probability. The second corresponds to a change in the current estimation technique in MC switching from the standard methodology, based on multiplying the number of electrons per particle contained in a certain spatial region by their velocity, to an approach based on the utilization of the Landauer formalism. The simulation results from both solutions were compared and contrasted to NEGF results from NESS, showing that the strategy based on the current computation modification using the Landauer approach constitutes the most suitable and reliable choice.

## References

[1] Medina-Bailon C., Padilla J., Sampedro C., Godoy A., Donetti L., Gámiz F. (2018). Source–to–Drain Tunneling Analysis in FDSOI, DGSOI and FinFET Devices by Means of Multi-Subband Ensemble Monte Carlo. IEEE Trans. Electron Devices.

[2] Medina-Bailon C., Carrillo-Nunez H., Lee J., Sampedro C., Padilla J.L., Donetti L., Georgiev V., Gamiz F., Asenov A. (2020). Quantum Enhancement of a S/D Tunneling Model in a 2D MS-EMC Nanodevice Simulator: NEGF Comparison and Impact of Effective Mass Variation. Micromachines.

[3] Wang J.W.J., Lundstrom M. Does source-to-drain tunneling limit the ultimate scaling of MOSFETs?. Proceedings of the Digest. International Electron Devices Meeting.

[4] Iwai H. (2015). Future of nano CMOS technology. Solid-State Electron..

[5] Berrada S., Dutta T., Carrillo-Nunez H., Duan M., Adamu-Lema F., Lee J., Georgiev V., Medina-Bailon C., Asenov A. NESS: New flexible Nano-Electronic Simulation Software. Proceedings of the 2018 International Conference on Simulation of Semiconductor Processes and Devices (SISPAD).

[6] Berrada S., Carrillo-Nunez H., Lee J., Medina-Bailon C., Dutta T., Badami O., Adamu-Lema F., Thirunavukkarasu V., Georgiev V., Asenov A. (2020). Nano-electronic Simulation Software (NESS): A flexible nano-device simulation platform. J. Comput. Electron..

[7] Medina-Bailon C., Badami O., Carrillo-Nunez H., Dutta T., Nagy D., Adamu-Lema F., Georgiev V.P., Asenov A. Enhanced Capabilities of the Nano-Electronic Simulation Software (NESS). Proceedings of the 2020 International Conference on Simulation of Semiconductor Processes and Devices (SISPAD).

[8] Medina-Bailon C., Dutta T., Adamu-Lema F., Rezaei A., Nagy D., Gergiev V.P., Asenov A. (2020). Nano-Electronic Simulation Software (NESS): A Novel Open-Source TCAD Simulation Environment. J. Microelectron. Manuf. Accept. Publ..

[9] (2018). QuantumATK Version O-2018.06. [Synopsys, Inc., 2018]. https://www.synopsys.com/silicon/quantumatk//.

[10] Sampedro C., Donetti L., Gámiz F., Godoy A. 3D Multi-Subband Ensemble Monte Carlo Simulator of FinFETs and Nanowire Transistors. Proceedings of the 2014 International Conference on Simulation of Semiconductor Processes and Devices (SISPAD).

[11] Sampedro C., Medina-Bailon C., Donetti L., Padilla J.L., Navarro C., Marquez C., Gamiz F. (2020). Multi-Subband Ensemble Monte Carlo Simulator for Nanodevices in the End of the Roadmap. Large-Scale Scientific Computations (LSSC).

[12] Venugopal R., Ren Z., Datta S., Lundstrom M.S., Jovanovic D. (2002). Simulating quantum transport in nanoscale transistors: Real versus mode-space approaches. J. Appl. Phys..

[13] Medina-Bailon C., Padilla J., Sadi T., Sampedro C., Godoy A., Donetti L., Georgiev V., Gámiz F., Asenov A. (2019). Multisubband Ensemble Monte Carlo Analysis of Tunneling Leakage Mechanisms in Ultrascaled FDSOI, DGSOI, and FinFET Devices. IEEE Trans. Electron Devices.

[14] Medina-Bailon C., Sampedro C., Padilla J.L., Godoy A., Donetti L., Gamiz F., Asenov A. MS-EMC vs. NEGF: A comparative study accounting for transport quantum corrections. Proceedings of the EUROSOI Workshop and International Conference on Ultimate Integration on Silicon (EUROSOI-ULIS).

[15] Carrillo-Nunez H., Ziegler A., Luisier M., Schenk A. (2015). Modeling direct band-to-band tunneling: From bulk to quantum-confined semiconductor devices. J. Appl. Phys..

[16] Datta S. (2000). Nanoscale device modelling: The Green’s function method. Superlattices Microstruct..

[17] Luisier M., Schenk A., Fichtner W. (2006). Quantum transport in two- and three-dimensional nanoscale transistors: Coupled mode effects in the nonequilibrium Greens function formalism. J. Appl. Phys..

